# Glyphosate and Aminomethylphosphonic Acid (AMPA) Modulate Glutathione S-Transferase in Non-Tumorigenic Prostate Cells

**DOI:** 10.3390/ijms24076323

**Published:** 2023-03-28

**Authors:** Dayanne Silva Borges, Lara Vecchi, Deysse Carla Tolentino Barros, Vinícius Marques Arruda, Helen Soares Valença Ferreira, Matheus Fernandes da Silva, Joyce Ferreira da Costa Guerra, Raoni Pais Siqueira, Thaise Gonçalves Araújo

**Affiliations:** 1 Laboratory of Genetics and Biotechnology, Institute of Biotechnology, Federal University of Uberlandia, Patos de Minas 38700-002, MG, Brazil; 2 Laboratory of Nanobiotechnology Prof. Dr. Luiz Ricardo Goulart Filho, Institute of Biotechnology, Federal University of Uberlandia, Uberlandia 38400-902, MG, Brazil; 3 Laboratory of Biochemistry, Institute of Biotechnology, Federal University of Uberlandia, Patos de Minas 38700-002, MG, Brazil

**Keywords:** prostate, organophosphate, glyphosate, aminomethylphosphonic acid, metabolism, transcripts

## Abstract

Glyphosate (GLY) was developed in the early 1970s and has become the most used broad-spectrum herbicide in the world so far. Its main metabolite is aminomethylphosphonic acid (AMPA), and the accumulation of GLY and its derivative compounds raises some concerns regarding possible health outcomes. In this study, we aimed to evaluate the effects of GLY and AMPA on prostate cell lines by evaluating cell viability, proliferation, gene and protein expression, and cellular pathways involved in the response to oxidative stress. Our results indicated that GLY and AMPA reduced the cell viability of tumorigenic and non-tumorigenic prostate cell lines only at higher concentrations (10 mM GLY and 20 mM AMPA). In contrast, both compounds increased the clonogenicity of non-tumorigenic PNT2 cells, mainly at concentrations below the IC_50_ (5 mM GLY and 10 mM AMPA). Moreover, treatment of non-tumorigenic cells with low concentrations of GLY or AMPA for 48 h increased GSTM3 expression at both mRNA and protein levels. In contrast, the treatments decrease the GST activity and induced an increase in oxidative stress, mainly at lower concentrations. Therefore, both compounds can cause cellular damage even at lower concentrations in non-tumorigenic PNT2 cells, mainly affecting cell proliferation and oxidative stress.

## 1. Introduction

Brazil is the fifth largest agricultural producer worldwide, being the main producer of coffee, sugarcane, and citrus [1]. Since agriculture is the leading contributor to the Brazilian economy, special attention must be paid to the use of synthetic chemicals and their economic, ecological, and health consequences [2]. While pesticides and herbicides have helped Brazil to become one of the top-producing countries, their use has raised several questions about their real safety. With an estimated world population of 8.5 billion in 2030, the demand for food is notorious, which highlights the need to fight weeds to increase agricultural production [3]. However, the excessive use of pesticides and herbicides results in the contamination of the soil and water, affecting the whole ecosystem [4].

Glyphosate (N-(phosphonomethyl)glycine-GLY) is a non-selective herbicide with broad-spectrum activity. It has been used worldwide in agriculture, forestry, and industry for weed control [5,6]. This compound interferes with the shikimate pathway in plants and microorganisms, thereby inhibiting the synthesis of aromatic amino acids [7]. GLY-based herbicides (GBHs) are the most widely used herbicide active ingredients in Brazil, with more than 200,000 tons sold in 2019 [8]. In the soil, GLY is degraded by microorganisms into its major metabolite, aminomethylphosphonic acid (AMPA) [9]. GLY and AMPA tend to accumulate in soils because of the inert nature of the carbon-phosphorus bond, which inhibits the enzymes responsible for its degradation. In addition, these compounds can adsorb to clay or organic matter, which further hinders degradation [10]. Dermal, oral, and respiratory routes are the most likely pathways through which GLY enters the human body [11,12], and GLY and AMPA were found in human serum samples after GLY intoxication [13,14]. There is no conclusive evidence about the metabolism of GLY into AMPA in the human body, but some authors have shown that traces of AMPA can be produced from GLY by the gut microbiota [15].

Although the European Food Safety Authority and the European Chemicals Agency concluded that GLY cannot be categorized as a carcinogen [16], GLY and its formulations were re-classified as probably carcinogenic to humans (i.e., Group 2A) by the World Health Organization’s International Agency for Research on Cancer (IARC) [17]. In fact, due to their alkylating abilities, GLY and AMPA induce double-stranded breaks in DNA, chromosomal aberrations, oxidative damage of nitrogenous bases, and alterations in the methylation patterns of oncogenes [18,19,20,21]. In this sense, they are associated with health outcomes such as solid tumors, non-Hodgkin lymphomas, endocrine disruption, and issues with the liver, heart, blood, and reproductive system [22]. Recently, Mesnage et al. (2022) demonstrated that GLY and its formulations activated DNA repair mechanisms in rats, increased the number of unfolded proteins, and triggered oxidative stress in the liver [23]. In addition, AMPA exposure was associated with increased breast cancer risk in a small cohort [24]. However, AMPA also showed an inhibitory effect against prostate cancer (PCa) cells in vitro [25,26] and inhibited PCa growth and metastasis in a xenograft mouse model [27].

Although environmental factors have been described to have a detrimental effect on prostate carcinogenesis [28], no study has demonstrated the effect of GLY and AMPA on the metabolism of prostate cells. PCa is the most common cancer and second leading cause of cancer-related mortality of men worldwide and in Brazil [29,30]. It is plausible that better understanding how GLY and AMPA modulate the molecular mechanisms of prostate cells might pave the way to understanding how these compounds can trigger cellular malignant transformation towards PCa development. Thus, the present study aimed to demonstrate the changes in gene expression and cellular pathways induced by GLY and AMPA in non-tumorigenic PNT2 cells. Our research meets the Sustainable Development Goals of the WHO, contributes to the fight against hunger (SDG 2), and promotes health (SDG 3), equality (SDG 10), and sustainability (SDG 11).

## 2. Results

### 2.1. GLY and AMPA Alter the Viability and Proliferation of Prostate Cells

**Figure 1:**
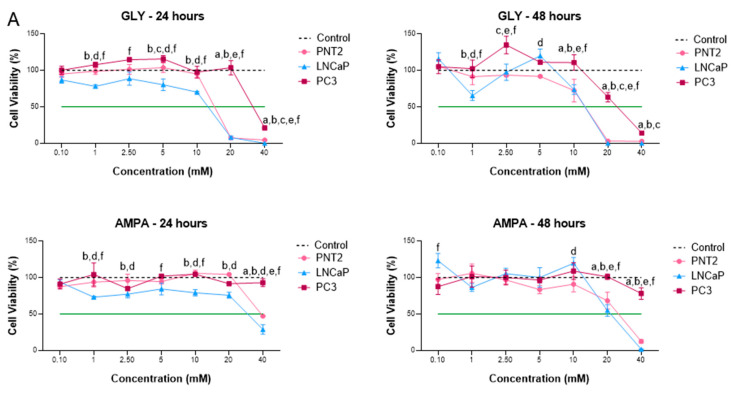

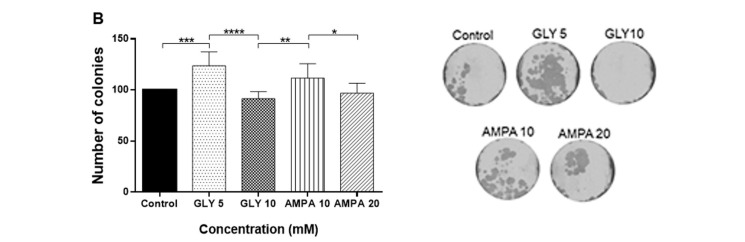
Effects of glyphosate (GLY) and aminomethylphosphonic acid (AMPA) on the viability and proliferation of prostate cell lines. (**A**) MTT assay was conducted for 24 and 48 h on PNT2 (non-tumorigenic), LNCaP (hormone-dependent tumorigenic), and PC-3 (hormone-independent tumorigenic) cell lines treated with GLY and AMPA. Lowercase letters over graph lines represent significant differences between cell lines: (a) PNT2 × Control (untreated cells), (b) LNCaP × Control, (c) PC-3 × Control, (d) PNT2 × LNCaP, (e) PNT2 × PC-3, (f) LNCaP × PC-3. The green-dashed line corresponds to 50% cell viability. (**B**) The colony formation assay was performed to evaluate the effect of GLY and AMPA on PNT2 cell proliferation (crystal violet staining). * *p* < 0.05, ** *p* < 0.01, *** *p* < 0.001, **** *p* < 0.0001. Results (**A**,**B**) are expressed as means ± standard deviation of three independent tests performed in triplicate.

At first, the cytotoxicity of GLY and AMPA was evaluated by the MTT assay for 24 and 48 h in non-tumorigenic (PNT2) and tumorigenic (LNCaP and PC-3) prostate cell lines (Figure 1). Remarkable cytotoxic activity was observed for GLY and AMPA after 48 h of treatment. Interestingly, PC-3 cells, which are hormone-independent and representative of a more aggressive stage of PCa, were more resistant and remained viable up to concentrations of 20 mM GLY for 24 h and 10 mM GLY for 48 h (Figure 1A). AMPA was less cytotoxic, and the viability of PC-3 cells remained similar to the control with untreated cells, even at higher concentrations (Figure 1A). For subsequent assays, the PNT2 cell line was chosen for the analysis of molecular changes mediated by GLY and AMPA in non-tumorigenic cells.

The concentrations that inhibited cell viability by 50% (IC_50_) were calculated (Table 1) and it was observed that LNCaP cells were the most sensitive. The IC_50_ of PC-3 cells was significantly higher than of the other cell lines, especially for AMPA, with an IC_50_ above the highest concentrations tested at 24 and 48 h. The behavior of PNT2 cells was similar to that of LNCaP cells. Regarding exposure to carcinogens as a risk factor for PCa, the IC_50_ values for the PNT2 (non-tumorigenic) cell lineage were then considered for subsequent assays. Two concentrations were chosen for treatment for 48 h, including values close to or below the IC_50_ for these cells. Thus, it was possible to observe the molecular effects that were not mediated by cytotoxicity but may be involved in the development of PCa.

The colony formation assay, used to evaluate the proliferative characteristics of in vitro cells, was performed to confirm the influence of GLY and AMPA on the clonogenic potential of PNT2 cells (Figure 1B). The number of cells colonies after treatment with 5 mM GLY was higher than the control (Figure 1). The results showed that GLY and AMPA affected the proliferative activity of PNT2 cells, so that lower concentrations (5 mM GLY and 10 mM AMPA) increased clonogenicity and higher concentrations (10 mM GLY and 20 mM AMPA) induced the opposite effect, with a decrease in the number of colonies. Importantly, the effects of GLY and AMPA diverged at the same concentration (10 mM), with more colonies observed when PNT2 cells were treated with AMPA.

### 2.2. GLY and AMPA Upregulate GSTM3 Transcripts in PNT2 Cells

The transcriptional levels of annexin A1 (*ANXA1*), cadherin 1 (*CDH1*), growth arrest specific 5 (*GAS5*), glutathione S-transferase mu 3 (*GSTM3*), interleukin 6 (*IL6*), transforming growth factor beta 1 (*TGFβ1*), and vimentin (*VIM*) were quantified to show the molecular events modulated by GLY and AMPA in the non-tumorigenic PNT2 cell line. Analyses were conducted comparing treated cells with controls (Table 2). *ANXA1* transcripts were upregulated 2.05-, 2.23-, and 3.97-fold when cells were treated with 10 mM GLY, 10 mM AMPA, and 20 mM AMPA, respectively. *GSTM3* mRNA expression was significantly upregulated 8.92-, 7.85-, 17.51-, and 34.86-fold when cells were treated with 5 mM GLY, 10 mM GLY, 10 mM AMPA, and 20 mM AMPA, respectively. Regarding *IL6*, gene expression was 2.54- and 3.02-fold higher in PNT2 cells treated with the lowest concentrations of GLY and AMPA. For *TGFβ1*, only AMPA altered its transcriptional levels by 2.05- (10 mM) and 2.30-fold (20 mM). *CDH1* expression was not altered ±2-fold and no *VIM* transcripts were detected. Therefore, we demonstrated for the first time that GLY and AMPA affected the oxidative balance of prostate cells by modulating GSTM3 expression.

### 2.3. Expression of GSTM3 and Enzymatic Activity in PNT2 Cells 

**Figure 2:**
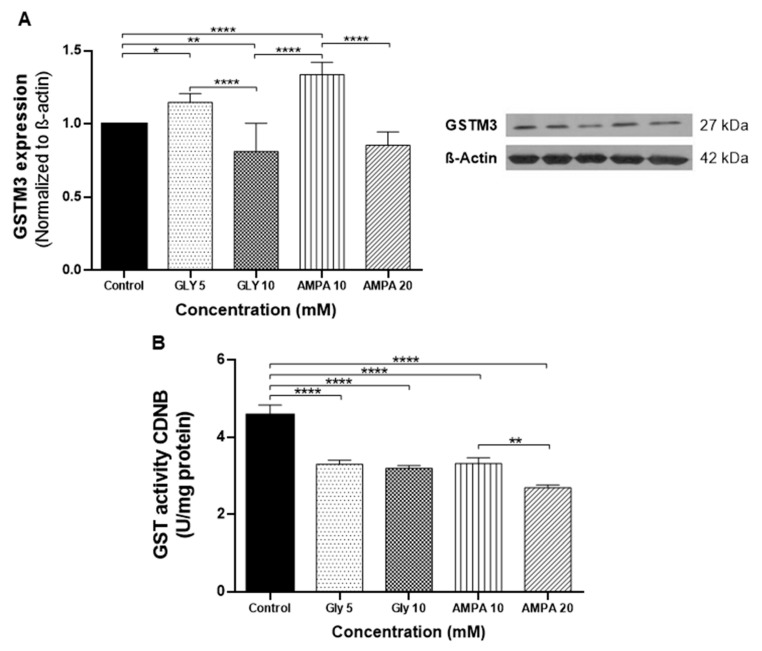
Glutathione S-transferase mu 3 (GSTM3) expression and enzymatic activity in PNT2 (non-tumorigenic) cells treated with glyphosate (GLY) and aminomethylphosphonic acid (AMPA) for 48 h. (**A**) The protein expression levels of GSTM3 were detected using western blotting. β-actin was used as the loading control. (**B**) Enzymatic activity of GST in cell homogenates: 3 µg of protein was analyzed in 0.1 M phosphate buffer, pH 6.5, with 0.1% Triton X-100, 0.1 M reduced glutathione (GSH), and 0.1 M 1-chloro-2,4-dinitrobenzene (CDNB) at 25 °C. Results are expressed as means ± standard deviation of three independent tests performed in triplicate. * *p* < 0.05, ** *p* < 0.01, **** *p* < 0.0001.

GSTM3 protein expression and GST enzymatic activity were evaluated in the PNT2 cell lineage after treatment with GLY (5 and 10 mM) and AMPA (10 and 20 mM) for 48 h (Figure 2). Treatment with GLY and AMPA at concentrations lower than the IC_50_ values (5 mM GLY and 10 mM AMPA) induced greater expression of GSTM3 compared to untreated cells and treatment with higher concentrations (10 mM GLY and 20 mM AMPA). GSTM3 expression was lower at higher concentration of the compounds (Figure 2A) compared to the controls.

GSTs are enzymes that act in the antioxidant defense system against synthetic compounds by catalyzing the conjugation of substrates to reduced glutathione (GSH), thereby reducing the liposolubility of the substance and facilitating renal elimination [31]. In this study, the detoxification activity of GSTM3 was evaluated through the enzymatic activity assay measuring the conjugation of 1-chloro-2,4-dinitrobenzene (CDNB) with GSH. GST activity was decreased in all treatments compared to untreated cells (Figure 2B). 

### 2.4. Effects of GLY and AMPA on Oxidative Stress and GAS5 Transcripts

**Figure 3:**
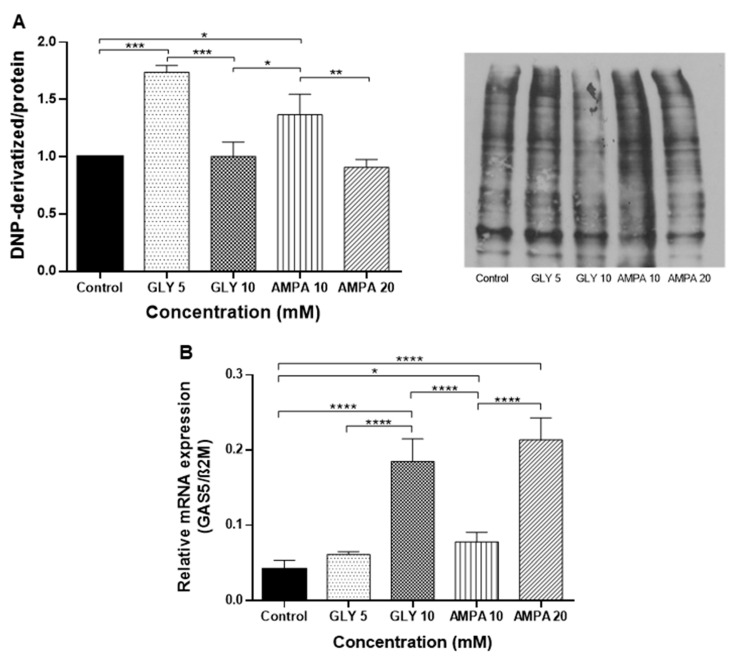
Protein oxidation assay and quantification of transcriptional levels of the growth arrest specific 5 (GAS5) in PNT2 cells. (**A**) Analysis of oxidized proteins from the PNT2 prostate cell line exposed to glyphosate (GLY) and aminomethylphosphonic acid (AMPA) for 48 h. (**B**) Expression of GAS5 by qPCR. β-2 microglobulin (β2M) was used as the reference. Results are expressed as means ± standard deviation of three independent tests performed in triplicate. * *p* < 0.05, ** *p* < 0.01, *** *p* < 0.001, **** *p* < 0.0001.

The Oxyblot assay was used to assess oxidative damage mediated by GLY and AMPA. The results demonstrate an increase in the levels of oxidized proteins in the PNT2 cells treated with lower concentrations of GLY (5 mM) and AMPA (10 mM), compared to untreated cells and treatment with 10 mM GLY and 20 mM AMPA. There were no significant differences between untreated cells and PNT2 cells treated with higher concentrations of GLY and AMPA (Figure 3A). Interestingly, treatment with the lower concentration of AMPA (10 mM) was responsible for a significant increase in oxidative stress compared to treatment with the higher concentration of GLY.

Finally, GSTM3 is regulated by lncRNA GAS5, a tumor suppressor that acts on tumor cells repressing proliferation, migration, and invasion, and promoting apoptosis and reactive oxygen species (ROS) [32]. Therefore, to verify whether the effects of the herbicides occurred at the post-transcriptional level, the expression of *GAS5* was quantified by qPCR. GLY (10 mM) and AMPA (20 mM) upregulated *GAS5* levels in PNT2 cells 4.42- and 5.11-fold, respectively. At lower concentrations, there was no change in *GAS5* transcripts by more than 2-fold compared to the control (Figure 3B).

## 3. Discussion

Carcinogenesis is a complex and multifactorial process that may be influenced by genetic, epigenetic, and environmental factors. An increase in the occurrence of many tumors is associated with exposure to environmental carcinogens, including pesticides and herbicides [33,34]. In this study, we sought to evaluate the mechanisms by which the herbicides GLY and AMPA alter the cellular signaling networks of prostate cells, focusing on aspects related to metabolism, and in particular, their effects on the antioxidant enzyme GSTM3.

GLY and AMPA are widely used by farmers worldwide, especially in Brazil, where the economy is based on agricultural production. Therefore, it is necessary to understand the impact of these compounds on the environment and their real contribution to the development of serious chronic diseases. After use, a fraction of GLY is absorbed by weeds and the rest is adsorbed in the soil, reducing its potential for action. In the soil, GLY is degraded by heterotrophic organisms to produce AMPA, its main metabolite [35]. Thus, our experimental design included reference substances of both GLY and AMPA since the possible damage induced by the chemical compounds could originate from the active ingredient, other constituents of the formulation, or a combination of both [36].

In this study, we first evaluated the cytotoxicity of GLY and AMPA against PNT2 (non-tumorigenic), LNCaP (hormone-dependent tumorigenic), and PC-3 (hormone-independent tumorigenic) prostate cell lines, with GLY presenting lower IC_50_ values. We also observed that the hormone-responsive tumor cell line was more sensitive to treatments, followed by the non-tumorigenic cells. Androgen-independent cells remained resistant and, after 48 h, their IC_50_ could not be calculated because it exceeded the value of the highest concentration used. This peculiar response may be associated with androgen responsiveness, as PC-3 cells represent hormone-independent prostate tumors. In fact, pesticides have been linked to endocrine disruption and the development of hormone-dependent cancers [37], including PCa [38]. However, according to the Endocrine Disruptor Screening Program (EDSP) of the United States Environmental Protection Agency [39] and reports from the European Food Safety Authority [40], the impact of GLY on endocrine disruption still needs to be widely investigated.

Li et al. (2013) also demonstrated that GLY and AMPA (15, 25, and 50 mM) inhibited cell growth and induced apoptosis in PCa cell lines with IC_50_ values above 40 mM for PC-3 cells, thus corroborating our results [25]. However, GLY cannot be considered as an anti-tumor compound since the herbicide was cytotoxic to tumor cells only at high concentrations. In addition, GLY can alter the viability of non-tumorigenic cells, which was already demonstrated by Abdel-Halim and Osman (2020) for the prostate cell lineage WPM-Y.1 and herein with PNT2 cells [41].

Notably, we observed that PC-3 cells treated with concentrations corresponding to the IC_50_ for PNT2 cells could proliferate or maintain their viability. Corroborating the hypothesis that herbicides can induce cell proliferation, we carried out colony formation assays with PNT2 cells exposed to GLY and AMPA at two concentrations: one corresponding to half of the IC_50_ value and another equivalent to the IC_50_. We verified an increase in the number of colonies after treatment with the lowest concentrations, which confirmed the MTT data and showed that even lower concentrations of these compounds can lead to relevant biological effects. Furthermore, when we considered the same concentration for GLY and AMPA (10 mM), the cellular response was different, with a proliferative effect observed for AMPA. Therefore, the analysis of both GLY and AMPA is important, as the cellular responses may differ depending on the concentration and time of exposure.

Dysregulation in gene expression is a key feature in the development of tumors, which directly affects different physiological mechanisms, such as adhesion, division, differentiation, angiogenesis, and cell death. Carcinogenic agents alter the molecular profile of non-tumorigenic or transformed cells, which may be related to the development and progression of cancer. Therefore, we evaluated the gene expression of *ANXA1*, *CDH1*, *GSTM3*, *IL6*, *TGFβ1*, and *VIM* after treatment of the PNT2 (non-tumorigenic) cell line with GLY and AMPA for 48 h. In these experiments, we observed that when PNT2 cells were treated with lower concentrations of the compounds, *ANXA1* and *TGFβ1* levels were significantly upregulated. *ANXA1* is a 38 kDa protein involved in signal transduction pathways, cell survival, proliferation, differentiation, migration, and development of different diseases, with pro- and anti-inflammatory roles [42,43]. In addition, it modulates the invasion, chemotherapy resistance, and aggressiveness of PCa [44,45] and inversely regulates the expression of *IL6* in prostate cells [46]. *TGF-β* also contributes to oncogenesis, increased proliferation, decreased apoptosis, epithelial-to-mesenchymal transition (EMT), and immune surveillance evasion [47].

However, the expression of *GSTM3* was the most significantly altered after treatment with GLY and AMPA. GSTM3 is a member of the GST family, which are cytosolic isoenzymes responsible for the detoxification of carcinogenic synthetic compounds [48]. With antioxidant potential, GST enzymes regulate stress-signaling pathways and have been associated with worse prognosis and chemoresistance in cancer. However, the data presented in the literature differ according to the type of tumor [49]. In PCa, there are no reports related to the expression of GSTM3, but polymorphisms in its coding gene have already been associated with predisposition to this disease [50]. For this reason, we also evaluated the expression of GSTM3 at the protein level and the activity of GST enzyme in PNT2 cells. At concentrations below the IC_50_, a higher expression of GSTM3 was observed. According to Checa-Rojas (2018), GSTM3 regulates the NF-κB and MAPK pathways, and we suggest that the upregulation of GSTM3 expression was responsible for the increased proliferation of PNT2 cells [49]. On the other hand, the GST enzyme activity decreased in all treatments, affecting the PNT2 response to oxidative stress, which was confirmed by the Oxyblot assay. In this respect, lower concentrations of GLY and AMPA led to greater protein oxidation. Interestingly, treatment with GLY and AMPA at the same concentration (10 mM) showed the enhanced effects of AMPA on oxidative stress.

Finally, in order to evaluate whether the herbicides could exert their effects at the post-transcriptional level, the *GAS5* transcripts were quantified. *GAS5* is a lncRNA described as a tumor suppressor and is negatively regulated in several cancers, such as bladder [51], liver [52], gastric [53], kidney [54], cervical [55], and PCa [56]. Furthermore, due to the regulatory nature of lncRNAs, *GAS5* was shown to be able to bind to GSTM3 in glioma cells [32]. In our study, *GAS5* transcriptional modulation was only observed in treatments at the highest concentrations of GLY and AMPA, which may be due to death mechanisms activated by values corresponding to the IC_50_. Therefore, the effects of these herbicides on the regulation of GSTM3 transcripts were not evident, highlighting their action on GSTM3 enzymatic activity.

Current knowledge about the cellular mechanisms associated with GLY is scarce, and there are not enough data to prove that GLY is metabolized into AMPA in humans or whether the AMPA detected in human samples originates from residues in the diet [57]. Lemke et al. (2021) suggested that only approximately 0.3% of GLY is metabolized into AMPA in humans, and GLY is mostly excreted in its original form through urine [58]. However, our data showed that alterations in cell metabolism were evident even at lower concentrations, especially for AMPA, which can affect pathways involved in the tumorigenesis process in prostate cells. Therefore, our study stands out for analyzing not only GLY, but also its metabolite, AMPA.

In summary, treatment of PNT2 cells with GLY and AMPA at concentrations below the IC_50_ increased the number of colonies formed as well as GSTM3 transcription and protein expression. However, the enzymatic activity was significantly reduced followed by a significant increase in oxidized protein levels. We suggest, therefore, that the herbicides inhibit the activity of an important protein related to the antioxidant response, leading to important metabolic alterations. As for the observed effects, GLY, and especially AMPA, increased oxidative stress by inhibiting enzymatic activity and inducing cell proliferation, despite the cellular feedback of increased transcription and GSTM3 protein levels. On the other hand, we believe that the results for the values equivalent to the IC_50_ were related to the activation of cell death induced by the high concentrations of the compounds. Our data highlight the pathways modulated by GLY and AMPA and demonstrate the need for further studies to understand their effects, even at trace concentrations.

## 4. Materials and Methods

### 4.1. Cell Lines 

Human prostate cell lines PNT2 (non-tumorigenic), LNCaP (PCa androgen-sensitive), and PC-3 (PCa androgen-independent) were purchased from ATCC. The cells were cultured in RPMI-1640 (Sigma-Aldrich, St. Louis, MO, USA) supplemented with 10% (*v*/*v*) fetal bovine serum (FBS, Gibco, ThermoFisher Scientific, Waltham, MA, USA) and 50 μg/mL gentamicin (Cultilab, Campinas, SP, Brazil). Cells were maintained at 37 °C in a humidified environment with 95% air and 5% CO_2_.

### 4.2. Chemicals

The compounds N-(phosphonomethyl) glycine glyphosate (GLY, molecular formula C**_3_**H**_8_**NO**_5_**P, CAS 107-83-6, purity ≥ 98%) and aminomethylphosphonic acid (AMPA, molecular formula CH**_6_**NO**_3_**P, CAS 1066-51-9, purity > 99%) were purchased from Sigma-Aldrich (St. Louis, MO, USA). All compounds for in vitro assays were reconstituted in complete culture media at 80 mM prior to use.

### 4.3. MTT 

Prostate cells PNT2 (1.0 × 10^4^ cells/well), PC-3 (1.0 × 10**^4^** cells/well), and LNCaP (1.2 × 10**^4^** cells/well) were seeded in 96-well plates and allowed to grow for 24 h. The culture medium was then removed and replaced with 150 μL of fresh medium containing GLY and AMPA solution at different concentrations (0.1, 1, 2.5, 5, 10, 20, and 40 mM). The compounds were diluted in RPMI medium with 10% (*v*/*v*) FBS. After 24 or 48 h of culture, MTT (5 mg/mL, Sigma-Aldrich, St. Louis, MO, USA) was added to the wells (4 h, 37 °C), followed by removal of the MTT solution and the addition of 200 μL/well of DMSO (Sigma-Aldrich, St. Louis, MO, USA) to solubilize the formazan. Absorbance was measured at 570 nm using a microplate reader (Thermo Plate, TP-Reader, Waltham, MA, USA). Percent cell viability was determined with respect to control.

### 4.4. Colony Formation 

PNT2 cells were seeded in 6-well plates at a density of 500 cells per well and incubated for 24 h prior to treatment. Then, the cells were treated with GLY (5 or 10 mM) or AMPA (10 or 20 mM) for 48 h, the medium was changed, and the cells were cultured for 15 days. Once colonies were formed, the cells were washed with phosphate-buffered saline (PBS) 1X, fixed with formaldehyde (4% *v*/*v*), and stained with crystal violet solution (0.5% *v*/*v*). The colonies were then photographed using L-Pix (Loccus Biotecnologia, Cotia, SP, Brazil), incubated with 300 µL of acetic acid (33% *v/v*), and 100 µL of this solution was transferred to a 96-well plate to evaluate the absorbance at 570 nm using a microplate reader (Thermo Plate, TP-Reader, Waltham, MA, USA). The quantitative determination of colony formation was calculated in relation to the untreated cells (considered as 100% colony formation).

### 4.5. qPCR Analysis

Total RNA was extracted from PNT2 cells treated with GLY (5 or 10 mM) or AMPA (10 or 20 mM) for 48 h using Trizol reagent (Invitrogen, Waltham, MA, USA), according to the manufacturer’s protocol. Samples were analyzed for integrity in 1.5% agarose gel and quantified and qualified by spectrophotometry using a Nanodrop 1000 (Thermo Fisher, Waltham, MA, USA). Then, first-strand cDNA was synthesized using M-MLV Reverse Transcriptase (Invitrogen) according to the manufacturer’s protocol. qPCR was performed using the StepOnePlus System (Applied Biosystems, Foster City, CA, USA) with Power SYBR Green PCR Master Mix (Applied Biosystems, Foster City, CA, USA), also according to the supplier’s instructions and using 0.5 µM of primers. The targeted genes were *ANXA1*, *CDH1*, *GAS5* [59], *GSTM3*, IL6, TGFβ1, and *VIM*, the expression levels of which were normalized to β-2 microglobulin (*β2M*) expression. The primers and amplicons are described in Table 3. The gene expression was calculated via the Cq comparative method after optimization of the standard comparative curve.

### 4.6. Western Blot 

PNT2 cells treated with GLY (5 or 10 mM) or AMPA (10 or 20 mM) for 48 h were lysed using NE-PER Nuclear and Cytoplasmic Extraction Reagents (Thermo Fisher Scientific, Waltham, MA, USA), according to the manufacturer’s protocol. Protein concentration was measured using the Bradford assay [60]. A 50 µg aliquot of protein was loaded per well in 12% SDS-PAGE gel, separated electrophoretically, and transferred onto a nitrocellulose membrane (GE Healthcare, Chicago, IL, USA). Afterwards, the membrane was blocked in 5% *(w*/*v*) skim milk powder in Tris-buffered saline with Tween (TBS-T), and then incubated for 16 h with primary antibody anti-GSTM3 (1:2000, Proteintech, Rosemont, IL, USA). Each blot was re-probed with anti-β-actin (1:3000, Sigma-Aldrich) used as a reference control. Then, blots were washed in TBS-T and incubated for 1 h in a 1:5000 dilution of peroxidase-conjugated secondary antibody (Sigma-Aldrich). Proteins were visualized using Luminata Classico Western HRP substrate (Merck Millipore, Burlington, MA, USA) and densitometry analysis of the band intensity was performed using ImageJ software (Version ImageJ bundled with 64-bit Java 8).

### 4.7. Enzymatic Activity

The enzymatic activity of GST was determined according to the method described by Habig and collaborators (1974), which is based on the conjugation of CDNB (Sigma-Aldrich) with reduced glutathione (Sigma-Aldrich) by enzymatic activity [61]. This conjugation is accompanied by an increase in absorbance at 340 nm, which is directly proportional to the amount of GST in the sample.

Briefly, PNT2 cells (3.0 × 10**^6^**) treated with GLY (5 or 10 mM) and AMPA (10 or 20 mM) for 48 h were collected using a cell scraper in a solution of 0.1 M phosphate buffer, pH 7.0, and 2 mM EDTA. Cell lysis was performed through 3 freeze/thaw cycles and the supernatant was collected after centrifugation for 15 min at 10,000× *g* at 4 °C.

The sample was subsequently added to the reaction mix containing 100 mM phosphate buffer, pH 6.5, with 0.1% Triton X-100 and 100 mM reduced glutathione in deionized water. To start the reaction, 10 μL of 100 mM CDNB in 95% ethanol was added. Absorbances were determined at 340 nm using a spectrophotometer every minute for 5 min. All analyses were performed in triplicate and the protein content was determined using the Bradford method (1976) [60] with bovine serum albumin as the standard. To calculate the GST activity, the absorbance delta per minute was used in the equation below:

GST specific activity:

$$\frac{\left(\Delta A_{340}\right)/\min\times \;V\;\left(\mathrm{mL}\right)\times \;\mathrm{dil}\;}{\varepsilon_{\mathrm{mM}}\times {\;V}_{\mathrm{enz}}\;\left(\mathrm{mL}\right)}=\;\mu \mathrm{mol}/\mathrm{mL}/\min\;$$

where:

dil = the dilution factor of the original sample.

ε mM (mM^−1^cm^−1^) = the extinction coefficient for CDNB conjugate at 340 nm for test in 1 mL cuvette = 9.6 mM^−1^ (path length^−1^ cm).

The result was expressed in U/mg of protein, where one unit was equivalent to the conjugation of 1 nmol of CDNB with reduced glutathione per minute.

### 4.8. Oxyblot Analysis

Protein carbonylation was assessed by measuring the levels of the carbonyl groups through the Oxyblot assay. Proteins from PNT2 cells treated with GLY (5 or 10 mM) or AMPA (10 or 20 mM) for 48 h were extracted as described above and 5 µg was denatured with 12% SDS. Carbonylated proteins were labeled by derivatization of the carbonyl group with 2,4-denitrophenylhydrazone (DNP) (10 mM) in 10% trifluoroacetic acid (TFA). After incubation for 20 min at room temperature, the proteins were neutralized with 2 M Tris and 30% glycerol. Proteins were loaded onto two identical 10% SDS-PAGE gels. One was stained with Coomassie Brilliant Blue R250 (Sigma-Aldrich) and the other was separated electrophoretically and then transferred to a 0.45 µm nitrocellulose membrane. Blocking was performed with 5% (*w*/*v*) skim milk in TBS-T 1X for 16 h at 4 °C. Subsequently, the membranes were stained with an anti-DNP antibody (1:1000, Santa Cruz Biotech) for 3 h followed by incubation with the secondary anti-rabbit IgG antibody (1:5000, Santa Cruz Biotech, Dallas, TX, USA) for 1 h. Protein visualization and evaluation of the pixel density of the bands were performed as described above.

### 4.9. Statistical Analysis

The data are represented as means ± standard deviation of three independent experiments performed (n = 3) in triplicate. Data normality was verified using the Kolmogorov–Smirnov test. One-way ANOVA followed by Tukey’s or Dunnet’s tests was performed to compare the means of different experimental conditions. The statistical significance was accepted when *p* < 0.05. GraphPad Prism v. 8.0 software (GraphPad, San Diego, CA, USA) was used to perform all the statistical analyses.

## Figures and Tables

**Table 1:** IC_50_ values for treatments of prostate cell lines with glyphosate (GLY) and aminomethyphosphonic (AMPA).

	*Cell Lineages–IC_50_ (mM)*					
	24 h			48 h		
*Herbicides*	PNT2	LNCaP	PC-3	PNT2	LNCaP	PC-3
*GLY*	15.15	13.04	37.59	11.54	10.48	22.85
*AMPA*	ND	30.54	ND	24.68	19.15	ND

ND: IC_50_ > higher concentration tested.

**Table 2:** Fold change in expression levels of mRNAs of different biomarkers in PNT2 (prostate, non-tumorigenic cells). Treatments were compared to untreated cells (control). Three independent experiments were performed (n = 3) in triplicate.

Gene	Treatment (mM)	RNA Expression	
		Fold Change	
		PNT2	*p* Value
*ANXA1*	Gly 5	1.34	ns
	Gly 10	2.05	***
	AMPA 10	2.23	***
	AMPA 20	3.97	****
*CDH1*	Gly 5	1.57	***
	Gly 10	1.46	**
	AMPA 10	1.42	**
	AMPA 20	1.12	ns
*GSTM3*	Gly 5	8.92	****
	Gly 10	7.85	****
	AMPA 10	17.51	****
	AMPA 20	34.86	****
*IL6*	Gly 5	−1.16	ns
	Gly 10	−2.54	****
	AMPA 10	1.11	ns
	AMPA 20	−3.02	****
*TGFβ1*	Gly 5	1.29	ns
	Gly 10	1.74	****
	AMPA 10	2.05	****
	AMPA 20	2.30	****
*VIM*	Gly 5	#	#
	Gly 10	#	#
	AMPA 10	#	#
	AMPA 20	#	#

Color key: 
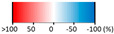
. # Vimentin was not expressed in PNT2 cells. ** *p* < 0.01; *** *p* < 0.001 and **** *p* < 0.0001. ns: non-significant. Annexin A1 (*ANXA1*), cadherin 1 (*CDH1*), glutathione S-transferase mu 3 (*GSTM3*), interleukin 6 (*IL6*), transforming growth factor beta 1 (*TGFβ1*), and vimentin (*VIM*).

**Table 3:** Sequences of primers used in qPCR reactions for the different targets. Annexin A1 (*ANXA1*), beta-2 microglobulin (*B2M*), cadherin 1 (*CDH1*), growth arrest specific 5 (*GAS5*), glutathione S-transferase mu 3 (*GSTM3*), interleukin 6 (*IL6*), transforming growth factor beta 1 (*TGFβ1*), and vimentin (*VIM*).

Gene	Sequence (5′-3′)	*Amplicon* (pb)
*ANXA1*	F: GATTCAGATGCCAGGGCCT R: CACTCTGCGAAGTTGTGGAT	110
*B2M*	F: CCTGCCGTGTGAACCATGT R: ACTGGGATATTCGTGGGCTG	94
*CDH1*	F: GTCATTGAGCCTGGCAATTTAG R: GTTGAGACTCCTCCATTCCTTC	97
*GAS5*	F: CTTGCCTGGACCAGCTTAAT R: CAAGCCGACTCTCCATACCT	82
*GSTM3*	F: ACTGGGATATTCGTGGGCTG R: CGCAAGATGGCATTGCTCT	214
*IL6*	F: GATTCCAAAGATGTAGCCGCC R: ATTTTCACCAGGCAAGTCTCCTC	242
*TGF-β1*	F: GTACCTGAACCCGTGTTGCTC R: CAGGAATTGTTGCTGTATTTCTGG	108
*VIM*	F: ACTAGAGATGGACAGGTTATCA R: GTAGGAGTGTCGGTTGTTAAG	218

## Data Availability

Not applicable.
